# Kidney Disease in HIV Infection

**DOI:** 10.3390/jcm8081254

**Published:** 2019-08-19

**Authors:** Gaetano Alfano, Gianni Cappelli, Francesco Fontana, Luca Di Lullo, Biagio Di Iorio, Antonio Bellasi, Giovanni Guaraldi

**Affiliations:** 1 Surgical, Medical and Dental Department of Morphological Sciences, Section of Nephrology, University of Modena and Reggio Emilia, 41125 Modena, Italy; 2 Nephrology Dialysis and Transplant Unit, University Hospital of Modena, 41125 Modena, Italy; 3 Department of Nephrology and Dialysis, “L. Parodi–Delfino” Hospital, 00034 Colleferro, Italy; 4 Department of Medicine, AORN “Antonio Cardarelli”, 80131 Naples, Italy; 5 Department of Research, Innovation, Brand Reputation, Ospedale di Bergamo, ASST Papa Giovanni XXIII, 24127 Bergamo, Italy; 6 Clinic of Infectious Diseases, Azienda Ospedaliero-Universitaria Policlinico di Modena, 41125 Modena, Italy

**Keywords:** CKD, chronic kidney disease, nephrotoxicity, HIV, antiretroviral therapy

## Abstract

Antiretroviral therapy (ART) has significantly improved life expectancy of infected subjects, generating a new epidemiological setting of people aging withHuman Immunodeficiency Virus (HIV). People living with HIV (PLWH), having longer life expectancy, now face several age-related conditions as well as side effects of long-term exposure of ART. Chronic kidney disease (CKD) is a common comorbidity in this population. CKD is a relentlessly progressive disease that may evolve toward end-stage renal disease (ESRD) and significantly affect quality of life and risk of death. Herein, we review current understanding of renal involvement in PLWH, mechanisms and risk factors for CKD as well as strategies for early recognition of renal dysfunction and best care of CKD.

## 1. Introduction

Despite epidemiological data reporting a reduction of new cases of HIV infection, its prevalence tends to augment globally because effective antiretroviral therapy (ART) has substantially increased survival of people living with HIV (PLWH), generating a new epidemiological setting of individuals aging with HIV [1]. 

Nowadays, life expectancy of highly educated PLWH treated chronically with combined ART has reached that of the uninfected counterpart [2]. The aging PLWH are therefore at risk for several age-related diseases including chronic kidney disease (CKD) [3]. Besides aging, several risk factors such as viral infection itself, ART, HIV-related comorbidities (e.g., diabetes mellitus, cardiovascular disease), coinfection (Hepatitis C Virus (HCV), Hepatitis B Virus (HBV), tuberculosis) and polypharmacy impact significantly on the development of kidney disease in this vulnerable population.

CKD is a relentlessly progressive disease that may evolve toward end-stage renal disease (ESRD). It contributes to poor quality of life and increases mortality of all affected subjects because it is associated with increased risk of cardiovascular disease, dyslipidemia, cognitive decline and bone disorders—comorbidities commonly found in PLWH, irrespective of their renal function [4]. Hence, superimposition of renal impairment on a complex disease such as HIV infection tends to increase the burden of comorbidities and, theoretically, predicts a worse outcome in this population. The best strategy to counteract the risk of CKD is based on prevention and early recognition of renal dysfunction as well as early initiation of ART in order to prevent long-lasting viral replication, which in turn is responsible for potential kidney damage [5]. The aim of this review is to provide a descriptive overview of the current understanding of CKD in the setting of HIV infection, focusing on prevalence, presentation, pathogenesis and treatment. Description of collateral issues such as comorbidities, coinfection and polypharmacy in PLWH who experienced CKD is out of the scope of this review.

## 2. Epidemiology of CKD in the Setting of HIV Infection

The measure of incidence and prevalence of CKD in the setting of HIV infection varies across geographic areas with large differences also within the same continent. Variability depends on a series of multiple factors such as methods to evaluate renal function, CKD definition, genetic heterogeneity, prevention program, access to health care system and initiation of combined ART. The first obstacle to overcome is the correct assessment of renal function since none of the methods used to estimated glomerular filtration rate (eGFR) have been validated in PLWH. The most widely used serum creatinine-based GFR estimating equations tend to be inaccurate in PLWH [6]. The major pitfall is the measurement of serum creatinine since it does not reflect true renal function in individuals with loss of lean body mass and malnutrition. Even cystatin C, an alternative marker of kidney function not-related to lean muscle mass, needs further studies before to be used in this population because it is influenced by HIV replication [7]. In addition, as in non-infected patients, it is unclear whether age-related decline in GFR reflects or not a physiologic process in this subset of population. 

Nuclear medicine method using 99mTc DTPA plasma clearance is the gold standard for GFR assessment, but it is impractical in resource-limited countries. In these settings, Chronic Kidney Disease Epidemiology Collaboration (CKD-EPI) [8]—or Cockcroft−Gault (CG) [9]—based eGFRs result in the most practical and economical methods to asses renal function.

The estimated prevalence of CKD in HIV population—measured using CKD-EPI equation [8]—ranges from 2.5 % in Europe to 7.4% in North America [10]. If we consider that 36.9 million of people are currently living with HIV worldwide [1], CKD represents a challenging problem with enormous implications for the national health care systems, probably not destined to be managed effectively in the next years given that the prevalence of CKD is expected to increase in this population [10]. PLWH have a slightly higher risk of developing CKD than HIV-uninfected subjects, but once CKD has commenced, the likelihood of developing ESRD is 2- to 20-fold greater compared to the uninfected counterpart [11,12]. The increased susceptibility is given by the combination of both traditional and HIV-specific risk factors for kidney disease. The first ones, commonly encountered in general population, include age, hypertension and diabetes [13]. The second are HIV replication, AIDS status, hepatitis B and C coinfection, low CD4 nadir, lipodystrophy and ART [10,14,15,16]. In a recent paper, Althoff et al. [17] elegantly estimated the population attributable fractions of preventable or modifiable HIV-related (CD4+ T-cell count and viral load) and traditional risk factors for several conditions including ESRD. The findings of this study showed that a substantial proportion of cases of ESRD could be prevented with interventions on traditional risk factors including hypertension, hypercholesterolemia, diabetes and smoking cessation. 

Epidemiological studies have revealed that patients of African descent have an 18 to 50% higher risk of developing HIV-related ESRD compared to whites [16,18,19]. This racial disparity is explained principally by a severe disease with rapid progression to renal insufficiency in people of African ancestry [16]. However, the risk of renal disease is not the same in all region of the African continent. Notably, West Africa has the highest prevalence of kidney disease [10]: 14.6% in studies using MDRD equation [20] and 9.2% in studies using CKD-EPI equation [8]. This unique susceptibility to developing renal disease is principally due to genetic factors. Techniques that examined ancestry informative markers have revealed that black patients, especially those living in West Africa and South Africa, carry certain polymorphisms that are linked with an aggressive course of HIV-associated nephropathy. In the last decade, risk alleles G1 and G2 of APO1, a gene sited on chromosome 22, have been strongly associated with the development of the most severe form of HIV-associated glomerulonephrites such as focal segmental glomerulosclerosis (FSGS) and HIV-associated nephropathy (HIVAN) [21,22]. Because up to 20–30% of patients of African origin have no APOL1 risk alleles, other variables may contribute to the profile risk in developing HIV-related nephropathy [23]. Among them, confounding environmental factors such as inadequate access to health care services and tendency of some national health policies to start ARTs at defined CD4 T-cell count cutoffs [24] need to be strongly considered because they may impact on the increased risk of CKD.

## 3. HIV Involvement in Kidney Disease

**Figure 1:**
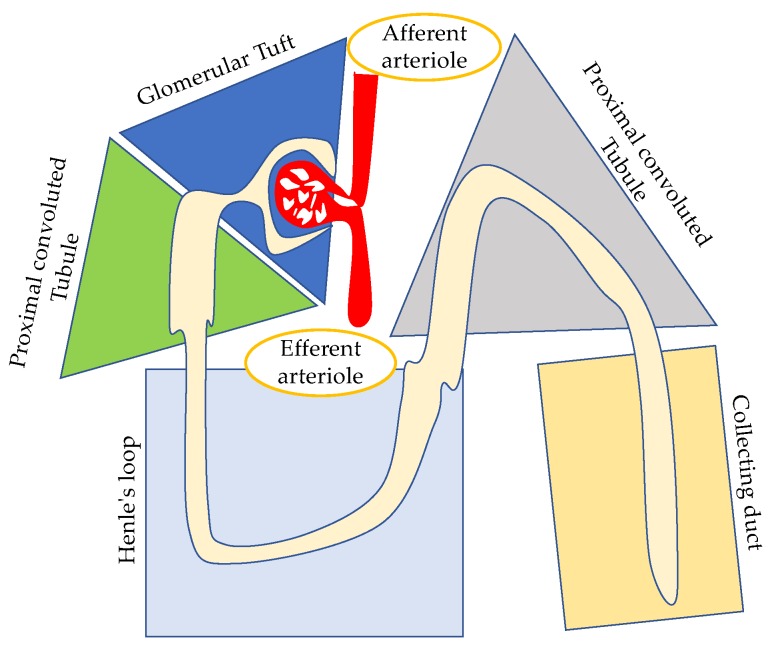
A schematic view of the nephron (functional unit of the kidney) and potential HIV involvement. Glomerular involvement has been described in HIV-associated nephropathy (HIVAN) (collapsing glomerulopathy); focal segmental glomerulosclerosis (FSGS), not otherwise specified; immune complex kidney disease. Tubulointerstitial involvement has been described as tubulointerstitial-dominant HIV-related diseases or secondary to antiretroviral agents that may cause direct renal injury manifesting with tubular dysfunction, acute interstitial nephritis and renal calculi. Vascular involvement called vascular-dominant HIV-related diseases such as thrombotic microangiopathy (TMA) syndrome, porpora thrombocytopenic (TTP), atypical hemolytic uremic syndrome (aHUS) have also been reported.

HIV infection is a chronic life-long illness which determines kidney damage essentially through a direct and indirect mechanism that may affect all structures of the nephron (Figure 1). The first is related to the cytopathic effect of the virus within the renal parenchymal cells resulting in the disruption of the normal cell activity. The second is not directly related to the pathological interaction of the virus with the infected cells, but relies on pseudopathological response of the immune system to HIV infections (e.g., formation of immune complexes depositing in the kidneys, hyperimmune reaction to HIV antigens), use of nephrotoxic drugs or other superimposed infections.

In order to offer a complete overview of renal disease in the context of chronic HIV infection, in February 2018 the Kidney Disease Improving Global Outcomes (KDIGO) has classified the nephropathies according to the tissue compartment involved [25] (Table 1). The classification reports also diabetic and age-related lesions diagnosed in the setting of HIV. 

Given the wide variability of renal pathologies, renal biopsy remains the best way to diagnose kidney disease accurately. To date, it is difficult to establish the likelihood of HIV as the etiologic factor of the aforementioned nephropathy because the identification of HIV antigens and specific anti-HIV antibodies within renal parenchyma is not practicable in the routine pathology laboratories. There is a reasonable grade of certainty only in classical HIVAN and in podocytopathies in HIV-infected newborns, whereas in all other cases causality is variable and is graded as high, medium and low.

According to the latest KDIGO classification, we describe histological, clinical features and principles of treatment of the most common nephropathies encountered in HIV settings. 

### 3.1. Glomerular-Dominant HIV-Related Diseases

HIVAN is a well-defined clinicopathologic entity manifesting as collapsing glomerulopathy associated with a consistent tubulointerstitial disease. The collapsed glomerulus is accompanied by dramatic hypertrophy and hyperplasia of the overlying glomerular epithelial cells, which tend to fill the urinary space [26]. IgM, C3 and C1q are generally detected in the collapsed segments and mesangial areas.

Tubules are focally enlarged (microcysts) and contain glass cast; often there is tubular atrophy, interstitial fibrosis and inflammation. At electron microscopy evaluation, the collapsed tuft is characterized by wrinkling of the glomerular basement membrane and hypertrophy of the overlying podocytes. Foot process effacement is observed both in collapsed and non-collapsed glomerulus. Classical HIVAN is strongly associated with APOL1 risk allele [27], South Africans [28] and low CD4+ T-cell count [29]. It manifests during acute HIV infection or AIDS-defining illness. Kidneys appear echogenic and often are enlarged on ultrasound imaging. Main clinical presentation is nephrotic proteinuria and rapid progression to ESRD. The mainstay of treatment for an ART-naïve patient is initiating antiretroviral drugs and avoiding potentially nephrotoxic agents. More importantly, ART may protect infected children from HIVAN, a disease associated with a tragic outcome in areas where there is a lack of facilities for renal replacement therapy (RRT) [30]. 

In the pre-ART era, several therapies were attempted to treat HIV-associated nephropathy. Observational studies showed that corticosteroid, the first-line therapy for non-HIV-related focal segmental glomerulosclerosis, was beneficial in the treatment of HIVAN [31,32,33,34]. 

In a retrospective cohort of 21 patients with biopsy-proven HIVAN, corticosteroid was administered at dosage of 60 mg for one month, followed by tapering over several months. Treated patients had a significantly slower progression of renal failure and improvement of proteinuria without an increased risk of infection, but relapse of proteinuria was common after reduction or steroid withdrawal [35]. Infectious Diseases Society of America (IDSA) guidelines [36] suggests treating HIVAN promptly with ART to reduce the risk of progression to ESRD. Angiotensin-converting-enzyme (ACE) inhibitors or angiotensin II receptor blockers should be used in the presence of stable renal function, and dose should be adjusted carefully in order to prevent hyperkalemia.

The strategy is to administer corticosteroid therapy in patients with progressive worsening of renal function despite optimal therapy with ART and angiotensin inhibition after exclusion of recent or subclinical chronic infections such as tuberculosis or viral hepatitis. In case of relapse, a further cycle of steroid is used in order to attain remission of proteinuria. Physicians should keep in mind that systemic corticosteroid may have notable infective complications (e.g., reactivation of herpes virus infections, tuberculosis, progression of Kaposi sarcoma and mucocutaneous candidiasis) as well as metabolic side effects (i.e., hyperglycemia, hypertension, osteoporosis and gastrointestinal ulceration) [37]. Therefore, a careful balance of the short-and long-term risks and potential benefits of steroid therapy should be undertaken before its use. 

Focal Segmental Glomerulosclerosis (FSGS), not otherwise specified (NOS), in the setting of HIV, is a virus-mediated attenuated form of FSGS. The main lesion is segmental sclerosis/segmental obliteration of capillary loops with matrix increase (with or without hyalinosis) within the glomerulus and adherence of capillary tuft to Bowman’s capsule [26]. Tubulointerstitial disease and the grade of podocyte effacement are generally less prominent than HIVAN. Different to HIVAN, it is more frequent in Caucasians than in African-Americans [27,38]. This variant is usually encountered in patients treated with ART with an undetectable viral load. 

It is unclear whether FSGS represents an identity different from HIVAN or lies in the same spectrum; it may be an attenuated histopathological pattern of HIVAN in patients treated with ART [27,38]. However, to confirm a presumed causality with HIV, other causes of FSGF should be excluded. Differential diagnosis of FSGS includes primary (idiopathic) FSGS, virus-associated forms (e.g., parvovirus B19 infection, SV40 infection, acute Cytomegalovirus (CMV) infection), drug induced-forms (e.g., interferon therapy, pamidronate toxicity) and forms mediated by adaptive structural-functional responses (e.g., hypertensive arterionephrosclerosis, diabetes, obesity). Treatment strategy is similar to that of HIVAN.

Immune complex kidney disease is a group of glomerulonephritis that includes IgA nephropathy (IgAN), lupus-like nephritis, membranous nephropathy and membranoproliferative glomerulonephritis. Technically, it is difficult to establish a clear causal link between these glomerulonephrites and viral infection; thus, the diagnosis of immune complex kidney disease in the setting of HIV remains plausible only when other secondary causes have been excluded. Clinical presentation is variable and depends on the location and extension of glomerular immune deposits. Generally, urinary sediment is active with dysmorphic red cells, there is a variable degree of proteinuria and the serum creatinine may be normal or elevated. Prognosis is better than HIVAN.

All glomerulonephritis but IgAN seem benefit from administration of ART [39]. If ART does not modify the course of glomerulonephritis ACE-Is or ARBs should be considered in relation to the grade of renal dysfunction. Immunosuppressive agents including steroids can be used in case of disease progression although this recommendation is not evidence-based as it derives from single clinical case reports and case series [40]. 

### 3.2. Tubulointerstitial-Dominant HIV-Related Diseases

Tubulointerstitial disease in the setting of HIV is due to classical HIVAN, drug toxicity or infections. 

Two forms are related to abnormal response of immune system to HIV: (i) diffuse infiltrative lymphocytosis syndrome (DILS) and (ii) immune reconstitution inflammatory syndrome (IRIS). The first is a hyperimmune reaction against HIV antigens. The second is an inflammatory syndrome directed against infectious antigens disseminated in the renal parenchyma, elicited by the recovery of immune system after starting of ART [41]. Both diseases are rare and are characterized by prominent interstitial CD8 T-cell infiltrates. 

Treatment of tubulointerstitial disease in the setting of HIVAN is described above, whereas management of drug nephrotoxicity and infectious disease is targeted to remove the offending agent and to treat the underlying infection, respectively.

First-line treatment of DILS is ART. Six to eight weeks of steroid are used to resolve severe organ dysfunction and allow rapid recovery of their function [42]. 

Management of IRIS is complex because phytopathology of the disorder is unknown. In the majority of cases, ART is continued and opportunistic infection is treated accordingly. Steroids may be needed for patients who develop severe kidney disease [37].

### 3.3. Vascular-Dominant HIV-Related Diseases

Vascular disease is the result of both direct and indirect effect of HIV infection on vessels. Although the virus is able to infect and dysregulate endothelial cells, multiple factors such as ART, HIV-associated comorbidities (i.e., dyslipidemia, chronic inflammations), traditional risk factors for arteriosclerosis (i.e., age, smoking, alcohol use) and nontraditional risk factors (i.e., hepatitis C, substance use or abuse) affect the development of vasculopathy. PLWH are prone to experience progressive atherosclerosis that involves pathological lipid metabolism and inappropriate activation of innate and adaptive immune systems in the arterial wall [43]. The process is so early that ART-naïve children without a significant burden or duration of risk factors for coronary heart disease have an increased carotid intima–media thickness (marker of atherosclerosis) compared to children without HIV infection [44].

Thrombotic microangiopathy (TMA) syndrome is a life-threating manifestation of HIV infection. The disease affects small blood vessels, and endothelial cell injury appears to be the primary event. It manifests with thrombocytopenia, microangiopathic hemolytic anemia, microvascular thrombosis and multisystem organ dysfunction [45]. The spectrum of renal injury ranges from mild to severe, sometimes requiring RRT [40]. The first step in the diagnosis of HIV-related TMA is rolling out infections and malignancies. These are common in patients with HIV/AIDS and can be themselves causes of purpura thrombocytopenic (TTP) or precipitating factors for atypical hemolytic uremic syndrome (aHUS). Treatment of TTP driven by HIV is based on the institution of ART, plasma exchange, and immune suppression (second-line therapy) [46]. Anti-C5 monoclonal antibody (eculizumab) is instead effective for the treatment of aHUS [47]. 

### 3.4. Other, in the Setting of HIV Infection

Diabetic nephropathy and arterionephrosclerosis may develop in PLWH given their multiple risk factors. HIV infection is associated with a four-fold increased risk of type 2 diabetes [48] and a poor glycemic control [49] compared to the uninfected counterpart. In a murine model, the expression of HIV-1 genes aggravates the progression of diabetic nephropathy, probably due to an upregulation of inflammatory citokines [50]. Arterionephrosclerosis is secondary to hypertension. Both diabetic nephropathy and arterionephrosclerosis manifest with proteinuria that may progress to nephrotic range in patients with long-term diabetes and severe hypertension.

Histological evaluation of kidney biopsy in diabetic nephropathy and arterionephrosclerosis shows both typical lesions of the underlying disease (e.g., Kimmelstein–Wilson nodules in diabetics and arterial wall medial thickening with arteriolar hyaline deposits in arterionephroslerosis) and sclerosis involving the perihilar region of the glomerulus. This latter histological pattern is compatible with a diagnosis of adaptive FSGS resulting from an excessive workload of the remaining nephrons. Treatment is directed toward the underlying cause. 

## 4. Nephrotoxicity of ART

WHO guidelines recommend a combination of three drugs to suppress HIV replication and prevent the development of drug-resistance strains. Therapy is generally based on two nucleos(t)ide reverse transcriptase inhibitors (NRTIs) (the “backbone” of ART) and a third agent from another class. 

The advent of ART has dramatically changed outcome of HIV+ patients in the last two decades reducing mortality and risk of progression to AIDS, even though important adverse side effects including nephrotoxicity arose with its use. Direct renal injury occurs with tubular dysfunction, acute interstitial nephritis and renal calculi. Indirect mechanisms of injury are due to medication dosing errors, drug–drug interactions, ART-induced rhabdomyolysis, lactic acidosis and metabolic complications.

Here we describe principal direct and indirect renal toxicity mediated by current antiretroviral drugs.

## 5. Direct Mechanism

### 5.1. Tenofovir

Tenofovir disoproxil fumarate (TDF) is a “preferred” nucleotide reverse transcriptase inhibitor (NRTI) widely used as part of antiretroviral therapy. Tenofovir is eliminated by renal elimination; dose-interval adjustments are mandatory in patients with renal impairment, even though its use is not recommended when GFR is less than 60 mL/min [36]. Nephrotoxicity of TDF has been extensively debated during the last decade because studies provided controversial results. Whereas clinical trial [51] and post-marketing report [52] did not report significant nephrotoxicity, observational studies [53,54,55,56,57] showed a concerning decline of renal function in a minority of patients [58]. Likely, the exclusion from trials of patients with CKD and the more frequent use of a less sensitive serum creatinine level than eGFR accounted for these differences. To shed light on this issue, a meta-analysis that included 10,889 participants showed that TDF-containing ART regimens were associated with a modest but significant loss of kidney function and a higher risk of acute renal injury compared to controls [59].

TDF causes kidney damage in 1–5% [52,60,61,62] of the cases, principally in patients with pre-existing renal disease, risk factors for CKD, concomitant use of potentially nephrotoxic agents, a low CD4 T-cell count and viral hepatitis B and/or C [63,64,65,66]. The risk of TDF-nephrotoxicity seems to increase with the concomitant use of other antiretroviral regimens; in particular, ritonavir-boosted protease inhibitor (PI/r) induces a greater decline in GFR than a non-NRTI agent, by increasing TDF exposure [57,67]. 

The mechanism underlying kidney damage is partially known, but it is likely related—as all NRTIs—to mitochondrial toxicity [68]. The main site of toxicity is the proximal tubule [69]. In a large cohort of 1202 patients, TDF increased the risk of proximal renal tubulopathy of about 3.3 fields compared with tenofovir-naïve patients [70]. Tubular dysfunction manifests with phosphaturia, hypophosphatemia and in an increase in serum alkaline phosphatase [71]. A complete Fanconi syndrome, characterized by a variable grade of glycosuria without diabetes, aminoaciduria, phosphaturia and renal tubular acidosis is instead uncommon in these patients. The most severe consequence of the loss of phosphate is an increased bone turnover manifesting with osteomalacia, a disease characterized by impaired mineralization in regenerating bone and pseudo-fracture around multiple joints [72]. 

Proximal tubular dysfunction is often overlooked because laboratory examinations show mild alterations, and initial presentation occurs without changes in kidney function; therefore, the diagnosis is often tardive and made during the worsening of renal function. TDF is able to induce distal tubular toxicity manifesting as nephrogenic diabetes insipidus [73] and distal renal tubular acidosis [74]. Compared to proximal renal tubular acidosis, determined by a defect in the ability to reabsorb bicarbonate, distal tubular acidosis is characterized by impairment in net acid secretion.

Tubular toxicity can lead to both a rapid and gradual reduction of renal function [75] which generally occurs during the first six months from its administration [67].

Acute renal failure has been reported between <1 and 2.2% [52,76,77]. There are conflicting data about reversibility of kidney injury after an episode of acute kidney insufficiency (AKI). It seems that renal dysfunction is only in part reversible [78,79,80]. A study conducted in 24 PLWH males, who stopped TDF for worsening of renal function, showed that renal injury was fully reversible only in 42% of subjects. Recovery of renal function occurred until the fifth month but the most improvement was documented during the first month after TDF withdrawal. The greatest GFR improvement was seen in patients with a rapid onset of AKI and a combining therapy with a PI/r. This suggests that a more rapid and, thus a more clinically recognizable, worsening of renal function is more likely to be fully reversible. Consequently, it is possible that the prolonged use of TDF causes an irreversible chronic tubule-interstitial fibrosis preventing the full recovery of the injury. However, the overall rate of discontinuation of TDF due to adverse renal events was lower than 2% [54,77,81].

Given the large use of TDF in ART regimen, all patients should be periodically evaluated (biannually) for tubular toxicity with analysis of serum phosphate level, and urine protein and glucose content [82]. This is particularly valid for patients with a combining therapy with ritonavir-boosted PIs. Data on use of TDF in patients with CKD are scarce and limited at one study in which the dose of the drug was inappropriately high for the grade of renal dysfunction. However, two of them (15.3%) experienced a concerning increment of serum creatinine level [77]. Dosage of beta 2-microglobulin, a marker of tubule damage, has been used to identify early patients who are at increased risk of kidney disease [83]. Nishijima et al. [84] documented for the first time that the measurement of beta 2-microglobulin within 180 days after initiation of TDF predicts the development of TDF-related nephropathy.

### 5.2. Tenofovir Alafenamide

Tenofovir alafenamide (TAF) is a novel prodrug of TDF recently approved for the first-line treatment of HIV+ patients. It is approved by Food and Drug Administration (FDA) as part of co-formulation and thus it is not available as single agent for the treatment of HIV infection. In the USA and Europe, TAF is usable as a stand-alone agent for treatment of chronic HBV infections. Studies comparing the pharmacokinetic properties of TAF and TDF have shown that TAF is able to reach a higher intracellular level in peripheral blood mononuclear cells than TDF, despite a lower concentration of the drug (about 90%) in the blood plasma [85,86,87]. Moreover, TAF has minimal mitochondrial toxicity compared to TDF in vivo studies [88]. Based on these findings, TAF-containing regimen leads to a more favorable renal and bone safety profile compared to TDF-containing regimen [89]. Two cases of TAF induced nephrotoxicity have been published to date, but it is difficult to ascribe the renal injury to TAF administration because more potential contributors were preexistent in those patients [90,91]. Further data are also necessary to evaluate the potential risk of TAF to induce Fanconi syndrome [92]. 

### 5.3. Protease Inhibitors

The protease inhibitors indinavir (IDV), lopinavir (LPV) and atazanavir (ATZ) have all been associated with renal stone formation [93]. IDV, an old drug responsible for significant formation of crystallization and stone formation in the urinary tract, is no longer used as antiretroviral therapy because has been replaced by newer drugs with a better side-effect profile [94].

Atazanavir is generally well tolerated and widely used with a boosting dose of ritonavir. Atazanavir has been associated with numerous case of crystalluria and nephrolithiasis [95]. Hamada et al. [96] found that ATZ had a hazard ratio of nephrolithiasis of 10.44 compared with other PI. ATZ withdrawal after the diagnosis of nephrolithiasis avoided new renal stone formation whereas continuum of the antiretroviral drug exposed the patients to recurrence of the disease [96]. Risk factors for calculi are dehydration, alkaline urine and a history of nephrolithiasis. Stones are radiolucent and are therefore not evident with abdomen X-rays [97].

### 5.4. Integrase Inhibitor

Raltegravir (RAL) and dolutegravir (DTG) are two antiretroviral drugs in the class of integrase inhibitors. According to recent WHO guidelines RAL and DTG are an alternative option to efavirenz for first-line ART [98]. Their renal effect is not insignificant as both drugs tend to increase serum creatinine concentration [99]. 

Metabolism of RAL occurs both in kidney and liver, but recommended dose remained unchanged in patients with CKD. Serum creatinine increase during the first 3–4 weeks of treatment without a tendency towards progression; a mean estimated creatinine clearance reduction of 5.4 mL/min was recorded after one year from its administration [100]. RAL causes an increase in serum creatinine without affecting tubular handling of creatinine. In fact, contrary to previous thinking, RAL does not block creatinine secretion through the inhibition of organic cation transporter 2 (OCT2) as DTG [99]. The mechanism underlying renal dysfunction is unknown and there are no data about the reversibility of the phenomenon to date. The decrease of cystatin C-based estimated GFR [101] may suggest a potential detrimental effect of RAL on renal blood flow or interstitial compartment. 

DTG does not need dose adjustment in CKD because it is excreted in urine for less than 1%. DTG augments plasma creatinine concentration thought inhibition of OCT2, a co-transporter of the basolateral side of proximal tubule cells responsible for urinary excretion of creatinine and drugs such as metformin and ranitidine. In this way, the unsecreted creatinine tends to increase in blood without an effective reduction of GFR due to drug-induced nephrotoxicity [102]. Similar to RAL, increase in serum creatinine level is not-progressive and occurs within the first four weeks of therapy [103]. The decline of CrCl is greater compared to RAL, 16.5 vs 5.4 mL/min at one year [100]. 

### 5.5. Cobicistat

The metabolism of cobicistat (COBI) is predominantly hepatic and dose adjustment is unnecessary in renal impairment. COBI has no antiviral activity against HIV. It acts, through inhibition of CYP3A, as a pharmaco-enhancer of atazanavir, darunavir or elvitegravir. This drug inhibits MATE1 (multidrug and toxin extruder protein1) a transporter located on the apical side of the proximal tubule responsible for the tubular excretion of creatinine. As a side effect, COBI increases serum creatinine levels and thus reduces eGFR without affecting true GFR [104].

## 6. Indirect Mechanism of Nephrotoxicity

About 50% of antiretroviral agents need dose adjustment in CKD. Inadequate dosing is common among CKD patients and it has been associated with treatment inefficacy, viral resistance and organ toxicity [105]. As recommended by FDA, drug dosing in renal dysfunction is based on creatinine clearance estimated by CG equations [9]. Unfortunately, this formula is inaccurate for extreme value of body weight and age. As a result, sarcopenia, malnutrition and ethnic differences impair true estimation of creatinine clearance and, thus, induce the physicians to make erroneous prescription of drugs. In this context, nuclear medicine techniques or 24-hour creatinine clearance [106] (for example, in low-income countries) may furnish valid support to dosing medications correctly.

Renal dysfunction can also result indirectly from drug interactions. The most common interactions in HIV-infected CKD patients occur principally with calcium channel blockers and statins. Drugs such as amlodipine, diltiazem, felodipine and nifedipine are metabolized by cytochrome P3A4, and as such their concentrations increase with concomitant administration of PIs, COBI (cytochrome inhibitors) and decrease with NNRTIs (cytochrome inducers). 

Regarding statins, simvastatin and lovastatin, both metabolized via CYP3A4, and should be avoided for the risk of severe myopathy and rhabdomyolysis. Fluvastatin, pitavastatin and pravastatin are preferable for their low potential of interactions. Atorvastatin and rosuvastatin are potent reducers of total LDL cholesterol, but their use is hindered by the moderate risk of drug-interactions and development of proteinuria, respectively [107].

Skeletal muscle involvement in HIV infection varies from asymptomatic muscle enzyme elevation to severe rhabdomyolysis. Zidovudine (ZDV) and abacavir may lead to myopathy, presumably through impairment of mitochondrial function. Rhabdomyolysis-induced AKI is a rare event and only few cases have been described in literature [108]. It occurs in patients with concomitant severe infection or in the context of substance abuse [109]. 

Didanosine (ddI), stavudine (d4T) and less frequently ZDV have been implicated in the pathogenesis of lactic acidosis. The mechanism underlying this condition is mediated by mitochondrial toxicity. Renal involvement manifests with acute renal failure, primarily due to prerenal causes (hypovolemia, reduced renal perfusion) [110]. 

ART determines a wide spectrum of metabolic alterations that may increase the occurrence of renal disease. All antiretroviral agents and protease inhibitors in particular are associated with cardiovascular disease that is mediated at least partly by dyslipidaemia [111]. Recently, the findings coming from a prospective cohort of the D:A:D study showed that darunavir, similar to the first-generation protease inhibitor, is associated with progressive increased risk of cardiovascular disease. Conversely, atazanavir lacks cardiovascular side effects [112].

The prevalence of diabetes mellitus rises constantly among PLWH with ART and is four times higher in infected patients with ART than in those uninfected [48]. Protease inhibitors and NRTIs are known to impair glucose tolerance, as well as predispose to frank diabetes mellitus. An increased rate of diabetic nephropathy has not been demonstrated but should be taken into account in case of albuminuria or proteinuria. 

In addition, some antiretroviral agents affect lipid profile and increase the risk of renal vascular disease [113]. Drugs like NRTIs (i.e., ddI, d4T, ZDV) are well-known to induce hypertriglyceridemia, whereas NNRTIs, mainly efavirenz, predisposed to hypertriglyceridemia and hypercholesterolemia. Abacavir instead increased the risk of cardiovascular diseases even in the absence of apparent lipid disorders [114].

## 7. Emerging Therapy

Doravirine (DOR) and bictegravir (BIC) are two novel antiretroviral agents recently approved by FDA for HIV treatment. Doravirine (DOR) is a recently approved NNRTI characterized by unique resistance profile, lack of food restriction and drug–drug interaction with antacids. DOR is a promising antiretroviral agent with the potential to become the first-line preferred regimen in naïve patients [115]. It does not require dose adjustment in patients with renal impairment nor when eGFR is less than 30 mL/min/1.73 m^2^ [116]. Bictegravir (BIC) is a newer INSTI acting without the need for a pharmacologic booster such as COBI or ritonavir (RTV). It is available only in the coformulation with TAF and emtricitabine (FTC). Since TAF an FTC need dose reduction in CKD this single-tablet regimen is not recommended in patients with moderate or severe CKD. The armamentarium of ART is expanding even with the use of biological therapies. Leronlimab (PRO 140) is a humanized antibody on the horizon. Target of leronlimab is CCR5, a receptor required for HIV-1 to enter macrophages, dendritic cells, and CD4 T-cells [117]. 

Regarding two-drug regimens, CKD patients with a suppressed viral load and no history of drug resistance have the opportunity to receive more tolerated and simpler maintenance therapy with DTG plus rilpivirine (RLP) compared to standard ART regimen [118]. More recently, the combination of DTG plus lamivudine (3TC) has been shown to be effective in treatment-naïve individuals [119].

## 8. Treatment of CKD

Treatment of CKD is based on blockade of the renin-angiotensin-aldosterone system and risk factor reduction. Diseases such as hypertension, diabetes, hyperuricemia, dyslipidemia and acidosis should be treated efficiently to prevent progression of renal disease. For patients who live in high-income countries and reach ESRD there are three options for treating uremia: hemodialysis, peritoneal dialysis and kidney transplantation. Patients on dialysis with a suppressed VL have survival rates comparable to dialysis patients without HIV infection, and choice of dialysis modality does not change outcomes. In low-income area, peritoneal dialysis is an alternative RRT option for ESRD patients because it is characterized by limited cost compared to HD. However, future studies are necessary to evaluate its safety since a high rate of peritonitis has been found among PLWH [120,121]. Kidney transplantation is the better option for HIV-infected individuals with ESRD requiring RRT [122]. Apart from a high rate of graft rejection, outcome of kidney transplant recipients is now similar to the uninfected counterpart [123]. 

In low-income countries as in Africa, treatment of CKD is hindered by limited numbers of nephrologists, limited resources, poor access to RRT and a high prevalence of poverty [124]. Here, there is no treatment of ESRD because dialysis is highly expansive and consumes a large proportion of the health budget. For this reason, country such as South Africa, have rationalized access to RRT only for patients who are suitable for transplantation [125]. Surprisingly, the prevalence of PLWN among individuals presented for dialysis is low compared to the proportion of HIV-infected individuals in the general population. This unexpected difference is due to the restrictive criteria of this program, indeed patients with uncontrolled HIV replication have no access to RRT [125]. Based on these data, early diagnosis and management of HIV would reduce the burden of health care cost associated with treatment of CKD and increase the number of PLWH that can be considered for dialysis and transplantation. In view of the advanced nature of presentation and lack of dialysis facilities, greater efforts are necessary to prevent early CKD. Screening programs for early diagnosis of kidney disease are needed at HIV detection as well as concurrent screening for diabetes and hypertension. 

## 9. Conclusions

HIV infection and CKD are two epidemic diseases with important social, clinical and economic implications. The interplay between these two diseases represents an interesting intersection between communicable and non-communicable diseases. Kidney disease and its associated risk factors are frequently found among HIV-infected patients, irrespective of the improvements with the use of ART, and it remains a cause of morbidity and mortality. The associated risk factors for kidney in this population include black race, older age, hypertension, diabetes, low CD4 T-cell count, and high viral load. The introduction of multi-drug combination therapy for the treatment of HIV has changed renal and overall outcome of PLWH over the last two decades. 

In the ART era, renal involvement is due principally to the toxic effects of ART and the multiple comorbidities of PLWH, even though a potential cytopathic effect of the virus may be also expected during ART with viral suppression [126]. Since the prevalence of HIV infection is high, nephrologists and HIV specialists will more frequently face patients with a variable grade of renal insufficiency and inevitably on RRT. The goal of their integrated multidisciplinary approach relies on close monitoring of renal function, mitigating the adverse effects of polypharmacy and avoiding inappropriate drug prescriptions.

## Figures and Tables

**Table 1:** Classification of HIV-related kidney disease and description of their treatment. Adapted from Swanepoel CR et al. “Kidney disease in the setting of HIV infection: conclusions from a Kidney Disease: Improving Global Outcomes (KDIGO) Controversies Conference” Kidney Int. 2018;93(3):545–559 (see reference [25]).

HIV-Related Kidney Disease	Directly Related to HIV Infection	First-Line Treatment *	Second-Line Treatment	Adjunctive Therapies
**I. Glomerular-dominant**				
a. Podocytopaties (e.g. HIVAN, FSGS)	X	ART	Steroid, Cys A	ACEi or ARB
b. Immune complex-mediated glomerular disease (e.g. MPGN, IgAN)		ART	Steroid	ACEi or ARB
**II. Tubulointerstitial-dominant**				
a. Tubulointerstitial injury in the setting of classic HIVAN	X	ART	Steroid	
b. Acute tubular injury or acute tubular necrosis (associated with ART)		Stop offending drug		
c. Drug-induced tubulointerstitial nephritis (other than ART)		Stop offending drug	Steroid	
d. Direct renal parenchymal infection by pathogens		Treat the underling infection		
e. Imuunologic dysfunction-related tubulointerstitial inflammation				
DILS IRIS		ART Treat the opportunistic infection/ART	Steroid Steroid	II. NSAID (risk of nephrotoxicity), thalidomide, hydroxychloroquine, anti-TNFalpha
f. Other tubulointerstitial inflammation in the setting of HIV		Treat underlining disease		
**III. Vascular-dominant**				
a. Thrombotic microangiopathy in the setting of HIV				
TTP aHUS	X X	ART, plasmapheresis ART, Eculizumab	I. Rituximab, bortezomib, Cys A	I. Sterodi/antiplatelet agents
b. Arteriosclerosis	X §	ART/reduce risk factors for atherosclerosis		
**IV. Other, in the setting of HIV**				
a. Diabetic nephrolopathy		Treat diabetes	ACEi or ARB	
b. Age-related nephrosclerosis		ART	ART/reduce risk factors for atherosclerosis	

Anti TNF-α, anti-tumor necrosis factor-α; ART, antiretroviral therapy; Cys A, Cyclosporine A; DILS, diffuse infiltrative lymphocytosis syndrome, FSGS, focal segmental glomerulosclerosis; HIVAN, HIV-associated nephropathy; IgAN, IgA nephropathy; IRIS, immune reconstitution inflammatory syndrome; MPGN, membranoproliferative glomerulonephritis; § Arteriosclerosis is due partly to cytopathic effect of HIV and to the traditional and non-traditional risk factors for vasculopathy; * According to current guidelines, ART is administered in all patients with the diagnosis of HIV infection regardless CD4 T-cell count. ART is indicated as the first-line approach when it is effective in the treatment of HIV-related kidney diseases.
